# Energy expenditure of bonobos across their locomotor repertoire

**DOI:** 10.1098/rsbl.2026.0427

**Published:** 2026-10-14

**Authors:** François Druelle, Jean-Pascal Guéry, Victor Narat

**Affiliations:** ^1^ UMR 7268 ADES, Aix-Marseille Université, CNRS, EFS, Marseille, France; ^2^ Functional Morphology Laboratory, Department of Biology, University of Antwerp, Antwerp, Belgium; ^3^ La Vallée des Singes, Romagne, France; ^4^ UMR 7206 EA, MNHN, CNRS, Université Paris Cité, Paris, France

**Keywords:** energetic, evolution, locomotor repertoire, point-mass model, *Pan paniscus*

## Abstract

Unlike quadrupedal mammals, primates use diverse locomotor modes adapted to arboreal environments. Such diversity of movements challenges allometric models, which underestimate energetic costs by focusing on steady-state walking. To address this, we combined ethological and biomechanical approaches to estimate the net metabolic cost of transport in bonobos (*Pan paniscus*). Using three-dimensional tracking and a point-mass model, we derived this estimate from net mechanical power and muscle efficiency across locomotor modes. Our results show that quadrupedalism in bonobos (10–12 J kg⁻¹ m⁻¹) exceeds mammalian predictions (approx. 3 J kg⁻¹ m⁻¹), whereas vertical ascent (26–32 J kg⁻¹ m⁻¹) is the most costly mode. However, arboreal locomotion incurs no major energetic penalty, given the high potential for energy recovery via kinetic/potential energy exchange during vertical descent and suspensory locomotion. By integrating these data with field-derived locomotor repertoires, we estimate that daily travel distances (0.4–6 km) account for 3–53% of bonobos’ daily energy expenditure. Reversing quadrupedal/bipedal proportions in this model shifts costs to 4–62%. Notably, even with 35% bipedalism, 2 km daily travel remains energetically feasible for bonobos. This suggests that early hominins could have incorporated bipedalism into diverse repertoires without facing energetic constraints, and that increased travel distance may have driven selective pressure for greater bipedal efficiency.

## 1. Introduction

Contrary to most quadrupedal mammals, primates exhibit a broad locomotor repertoire (including walking, scrambling, running, leaping, dropping, climbing, suspensory locomotion, brachiation and dynamic transitions between these modes) that evolved for arboreal life (e.g. [1–7]). The arboreal environment is inherently discontinuous, variable, compliant (i.e. characterized by branch flexibility and deflection) and instable, challenging the mechanical requirements of legged locomotion, including propulsion, balance control, stability and manoeuvrability. Consequently, primate locomotion is non-steady, showing high variability in acceleration, intensity and duration, directly influencing muscle contraction rates and energy expenditure [8–13]. Even modest perturbations, such as changes in direction and speed, or walking on uneven terrain, increase metabolic rate [13], whereas intense activities such as climbing, jumping and tree swaying dramatically raise it [14]. Therefore, dynamic arboreal movement with balance control and grasping is more energetically demanding than steady travel on continuous, horizontal ground. In this context, elevated muscular activity and co-activation are probably common in primates, particularly in large species like great apes.

General allometric equations and energetic models have provided valuable insights into locomotor costs. Yet, these models are typically based on respirometry studies that measure the average energy expenditure of steady-state locomotion (walking, running, climbing) under highly controlled conditions [15–18]. This approach relies on a stoichiometric assumption to infer adenosine triphosphate (ATP) energy yield from oxygen consumption, which is then used to estimate muscle ATP energy consumption. It requires prolonged, constant-intensity activity to achieve equilibrium between oxygen uptake and cellular oxygen consumption. Although such measurements are appropriate for broad inferences about quadrupedal terrestrial mammals [14,17,19], primate locomotion, including a continuous scheme of movements (see discussion in [5]) lacking clear boundaries and involving smooth transitions between certain modes [20], deviates from these idealized conditions. The constant need to shift between locomotor modes in complex arboreal environments complicates accurate energetic assessments using respiratory gases. This is due to the temporal delays and physiological processes, such as the lag between muscle force production and oxygen consumption measured at the mouth (e.g. [11,21,22]) that disrupt the link between oxygen uptake and ATP turnover. Although wearable tracking devices, such as accelerometers, offer effective energy expenditure proxies (e.g. [23–25]), their use in non-human primates remains limited owing to ethical and practical constraints, such as device self-removal by the animals or their congeners.

In this context, walking-based cost estimates probably underestimate primate energy expenditure [15,26]. We hypothesize that dynamic three-dimensional (3D) movement, that is locomotion involving changes in direction, acceleration and gait, incurs higher net energetic costs than steady, terrestrial movement. To test this, we integrate two traditionally separate approaches: (i) ethological studies, which quantify locomotor repertoire (mode, substrate, context) and show primates as generalists (e.g. [27–30]) with poorly understood energetic implications and (ii) biomechanical analyses, which measure mode-specific performance (e.g. [31–37]), but rarely address natural movement sequences (but see [14,38–41]). By linking these frameworks, we estimate net energetic costs of transport (J kg^–1^ m^–1^) across major locomotor modes, weighted by frequency and distance, to quantify overall locomotor costs in bonobos (*Pan paniscus*). As a large-bodied, arboreal primate and a relevant last common ancestor model (e.g. [42–44]), bonobos allow us to explore how changes in bipedal locomotion proportion within their locomotor repertoire affect overall energetic costs.

Using 3D tracking and a point-mass model, we estimate external mechanical work (i.e. the energy used to move the body through space, such as raising and accelerating it (positive work) or lowering and decelerating it (negative work)) for locomotor modes. Although metabolic cost cannot be directly measured and external work accounts for only part of it (e.g. [45,46]), adding muscle efficiency estimates (19%–35% for positive power [45]; –100% and –120% for negative power; e.g. [47–50]) should approximate net metabolic cost. This yields a range of values for bonobos, allowing comparison of energetic costs and energy recovery (via kinetic-potential energy exchange, as described by the ‘inverted pendulum’ mechanism; e.g. [51–54]) across modes. In addition, we evaluate literature-reported locomotor repertoires [55–57] and model a hypothetical transition towards more bipedal walking, hypothesizing that quadrupedal walking is less costly than arboreal displacement and that increased bipedalism would result in unsustainably high daily costs.

## 2. Material and methods

### (a) Study site

A group of 17 bonobos (11 females, 6 males; 11 matures >12 years of age, 6 immatures <12 years of age) was recorded at the zoological park La Vallée des Singes (France) during three full-day sessions in March 2022. Recordings took place in the group's outdoor enclosure, which consists of an approximately 7000 m^2^ island covered with various vegetation, including trees and bushes [58].

### (b) Video recording

Locomotor sequences were captured using a multi-camera setup of four GoPro Hero8 black cameras (120 fps, 1920 × 1080 pixels resolution), each fixed to rigid supports (tree trunks or barriers) using articulated arms. Cameras were oriented towards the same area and were triggered roughly synchronously via a Bluetooth remote control. Short locomotor sequences, each lasting a few dozen seconds, were filmed. In total, 69 sequences were kept for the analysis, including sequences of movement with quadrupedal walking and running, bipedal walking, vertical ascent and descent, leaping and torso-orthograde suspensory locomotion (see [3]; figure 1; the distribution of selected locomotor bouts across age classes is available in the supplementary material, A). Given the methodology used, including the large field of view and the distance from the bonobos, individuals were mainly unidentifiable (see figure 1), and neither age nor sex was considered in the present analysis.

**Figure 1.**
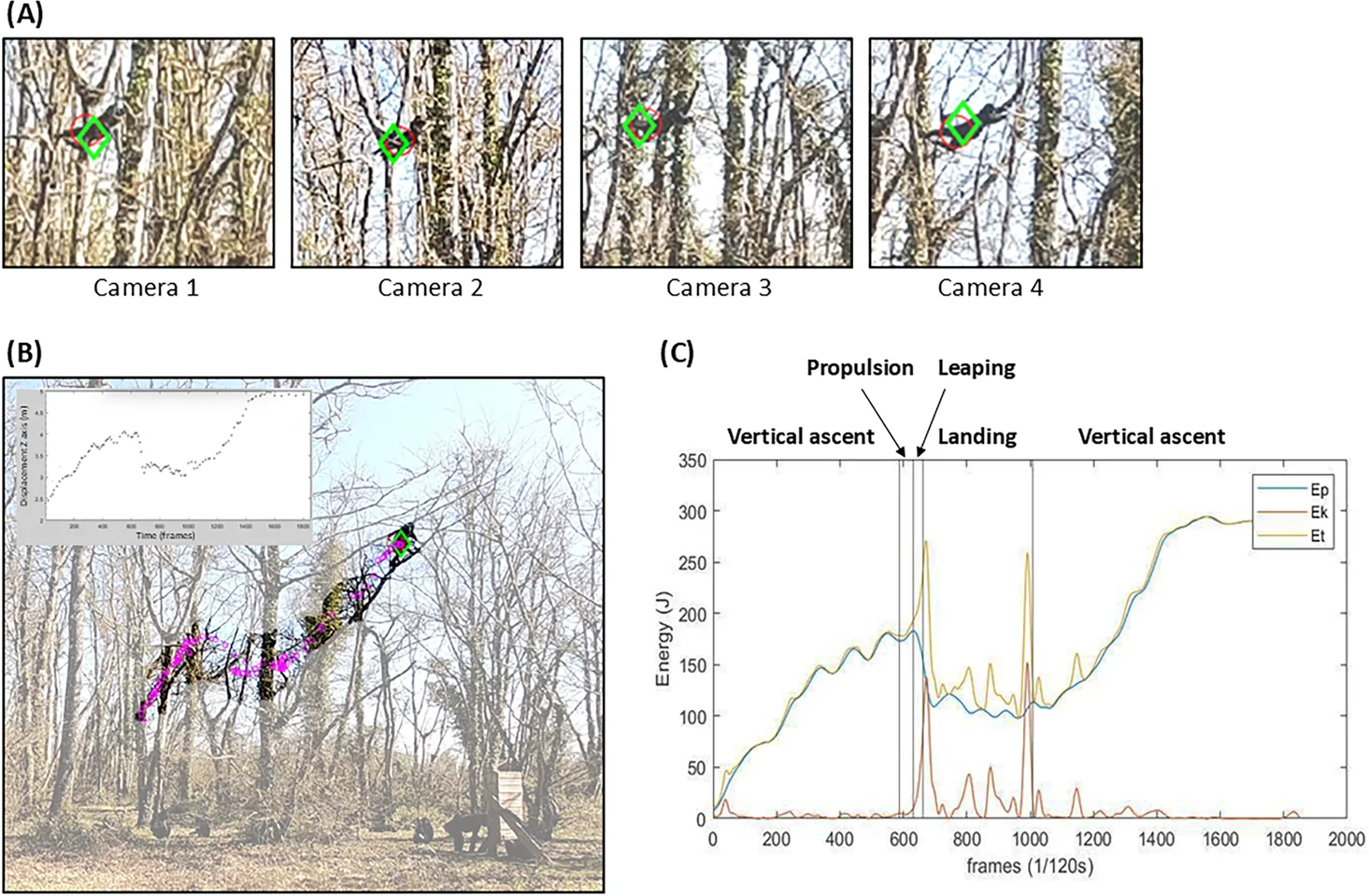
Overview of the bonobo protocol. (A) Simultaneous video stills (zoom-in views) of a leap from four GoPro cameras; green diamond (generated by DLTdv8) shows the calibrated corresponding point. (B) Full locomotor sequence digitized in 3D (pink open circles) with vertical displacement (*z*-axis). (C) Kinetic (Ek), potential (Ep) and total energy (Et) variation throughout the sequence.

### (c) Calibration and 3D reconstruction

The recording volume was calibrated each morning before the bonobos entered the enclosure. Two areas were defined: one open area measuring 23 × 11 × 4 m (length × depth × height) and a closer and vegetated area measuring 13 × 6 × 10 m. Calibration was performed using a custom wand (two tennis balls fixed to the ends of a rigid stick measuring 1.5 m). To ensure accurate temporal calibration, we used photographs and subsequently compiled them into a single movie sequence per camera (see also [59]). Seventy calibration photographs were taken per camera in the open area during a 1-day recording session, and 273 and 138 calibration photographs were taken per camera in the more densely vegetated area during 2-day recording sessions. Calibration data were processed using easyWand5 software [60] to generate direct linear transformation (DLT) coefficients for DLTdv8 application [61], with calibration errors (i.e. wand endpoint standard deviations) of 12 mm (open area), and 28 mm and 16 mm (closed area).

### (d) Digitization and mechanical analysis

Bonobo movements were manually digitized at the level of the hip joint in the reconstructed volume, allowing a kinematic analysis of locomotor sequences. Recordings began when a bonobo initiated movement and ended when locomotion ceased, whereas the animal remained within the calibrated volume. The total mechanical energy (TE; or centre of mass mechanical energy) derives from the motion of the estimated centre of mass. The hip joint served as a proxy, as the centre of mass remains proximal to it across diverse postures (see supplementary material, B, for sensitivity analysis).

Using MATLAB (R2025a), we first applied a general filter using linear interpolation to address missing data. We then applied a fourth-order zero-phase-shift Butterworth low-pass filter with a cut-off frequency of 4 Hz to reduce the inherent noise associated with manual digitization. The rationale for this cut-off frequency, along with its evaluation, is detailed in supplementary material, C (see also figure 1B). Linear velocities were obtained via finite difference calculus. The specific TE of this point-mass was computed as the sum of specific gravitational potential energy (PE) and specific kinetic energy (KE), as follows:

$$\begin{array}{rl} & \;\;Gravitational\;PE:\;PE\;=\;g\Delta Z,\\ & Translational\;KE:\;KE\;=\frac{1}{2}\;\left({\dot{X}}^{2}+{\dot{Y}}^{2}+{\dot{Z}}^{2}\right),\\ & \;\;\;TE:\;TE\;=\;PE+KE, \end{array}$$

where *g* is the gravitational acceleration (9.81 m s^−2^), ΔZ is the height of the body starting at 0 at the origin of the movement sequence (i.e. the horizontal reference axis), and *X* and *Y* are the position of the point-mass in the horizontal plane (perpendicular to the plane defined by the gravitational vector; see figure 1C). The time derivative of work yields the instantaneous power fluctuations of the point mass.

Instantaneous metabolic power can be estimated using different efficiency values for positive and negative work. Muscular efficiency varies by work type: positive work consumes more metabolic energy than negative work (e.g. [7,9,47,50]), and may also vary among species and locomotor modes (e.g. [45,51,62]). Maximum muscular efficiency for converting chemical energy into positive mechanical work is typically around 0.25 [63], with higher apparent efficiency values probably owing to energy elastic storage via tendons and other passive structures (e.g. [51,62]). Negative work costs are approximately one-fifth to one-sixth of positive work [47,51], implying a higher efficiency for eccentric contractions. Here, we used two approaches to estimate net metabolic power:

— Fixed efficiency values; (i) 0.25 for positive work and –1.20 for negative work. (ii) A lower net efficiency of 0.20 for positive work and –1 for negative work.
— Mode-specific values; for walking (tripedal, bipedal and quadrupedal), we used a net efficiency of 0.19 for positive work, estimated for chimpanzees based on Heglund *et al.* [64] and the net cost of transport reported in Pontzer *et al.* [65]. For vertical ascent, leaping and running, we adopted a net efficiency of 0.24 for positive work (see [66]). For suspensory locomotion, we assumed a higher net efficiency of 0.35 for positive work, as this mode probably allows for optimal elastic energy exchange, similar to human walking [45]. For negative work, we used a fixed cost of –1.20 across all modes.

The time integral of the total instantaneous power yields an estimate of the metabolic energy costs for the considered locomotor bout within the sequence of movement. We calculate the cost of transport by dividing the total energy spent during a locomotor bout by the distance travelled.

We estimated the percentage of pendular energy recovery of the BCoM in different modes of progression [64]. It is estimated as follows:

$$\begin{array}{r} Recovery\;\left(\%\right)=\frac{\Delta^{+}PE+\Delta^{+}KE-\Delta^{+}TE}{\Delta^{+}PE+\Delta^{+}KE}\;\times 100, \end{array}$$

where$\;\Delta^{+}PE$, $\Delta^{+}KE$ and $\Delta^{+}TE$ are the sum of the positive increments of the specific potential, specific kinetic and specific total energy, respectively.

We tested the relationship between the speed of progression across the different locomotor modes and both the energy recovery and net metabolic cost of transport using Pearson correlation tests in StatXact. We evaluated the overall energetic costs of the locomotor repertoires documented for bonobos by weighting the average net metabolic cost of transport for each mode by its frequency and its average distance travelled, and summing the resulting values across all modes.

## 3. Results and discussion

### (a) Estimating locomotor performance and energy expenditure

Positive and negative work are balanced in horizontal ground locomotion but unbalanced in arboreal modes (vertical ascent, vertical descent, suspensory locomotion and leaping; figure 2A). Net estimated metabolic cost of transport correlates strongly with speed during bipedal and tripedal walking (*r* = 0.66–0.67 and *r* = 0.91, *p* < 0.05, respectively; figure 3A–C), but not quadrupedal walking, which shows two contexts: steady open-area walking (6–7 ± 1 J kg⁻¹ m⁻¹), and variable vegetated-area walking (14–18 ± 7–8 J kg⁻¹ m⁻¹). Applying the two general equations used by Pontzer & Wrangham [67] to calculate the energetic cost of terrestrial travel yields estimates very similar to our initial value for an average walking speed of 1 m s⁻¹: 7.3 J kg⁻¹ m⁻¹ (according to the ‘Primates’ equation [16]) and 9.4 J kg⁻¹ m⁻¹ [15]. Overall, our estimates thus align with literature, exceed mammalian predictions (approx. 3 J kg⁻¹ m⁻¹ [16]), and mirror chimpanzee treadmill data using respirometry protocols (5 J kg⁻¹ m⁻¹ [65]). This reinforces the view that great apes walk uneconomically compared with non-primate quadrupedal mammals.

**Figure 2.**
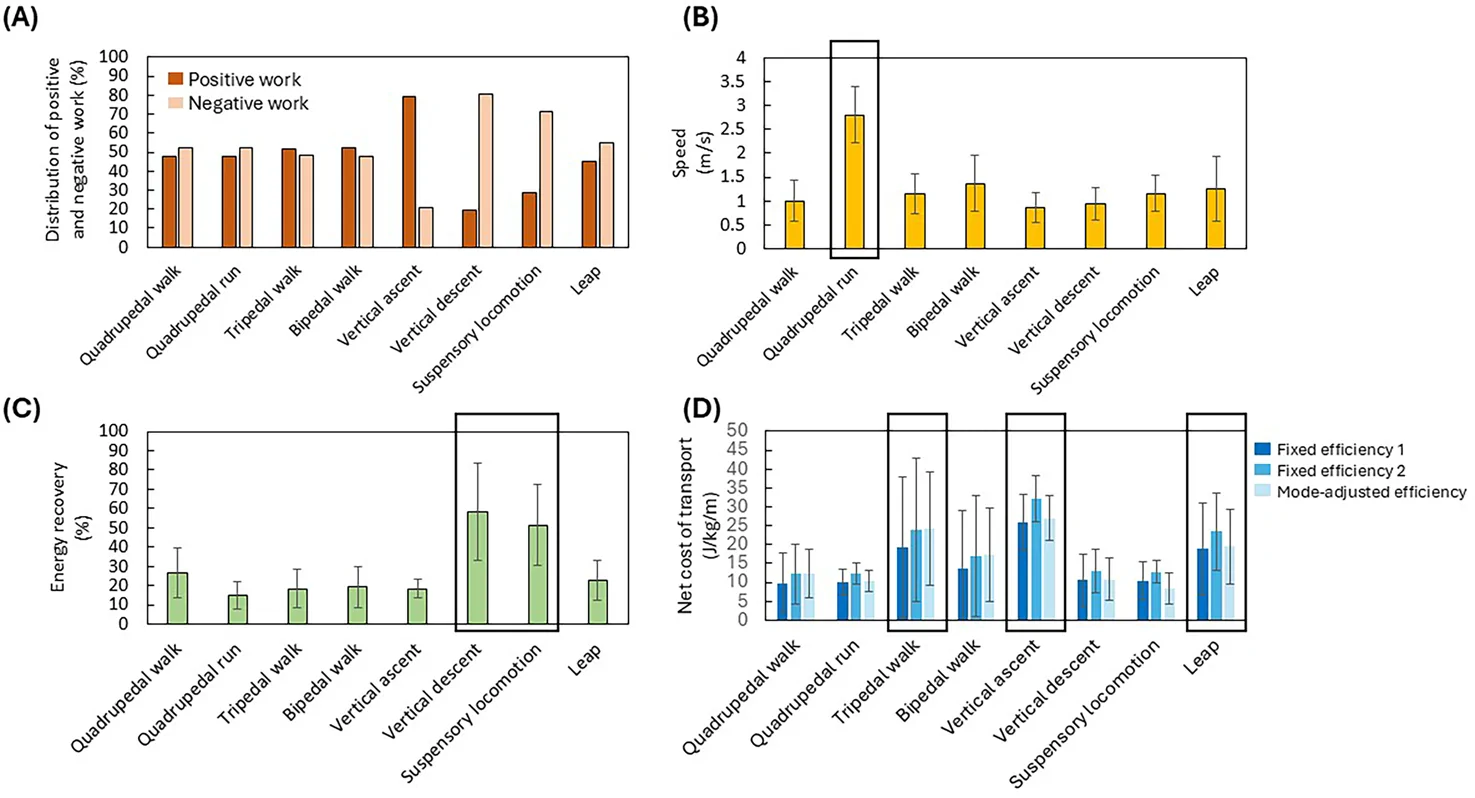
Average performance variables across locomotor modes, with error bars indicating standard deviation. (A) Distribution of positive (darker) and negative (lighter) work; (B) average speed; (C) average energy recovery; (D) net estimated metabolic cost of transport with fixed efficiency values 1 (dark blue), 2 (medium blue) and mode-adjusted values (light blue). Squares indicate modes with higher average values.

**Figure 3.**
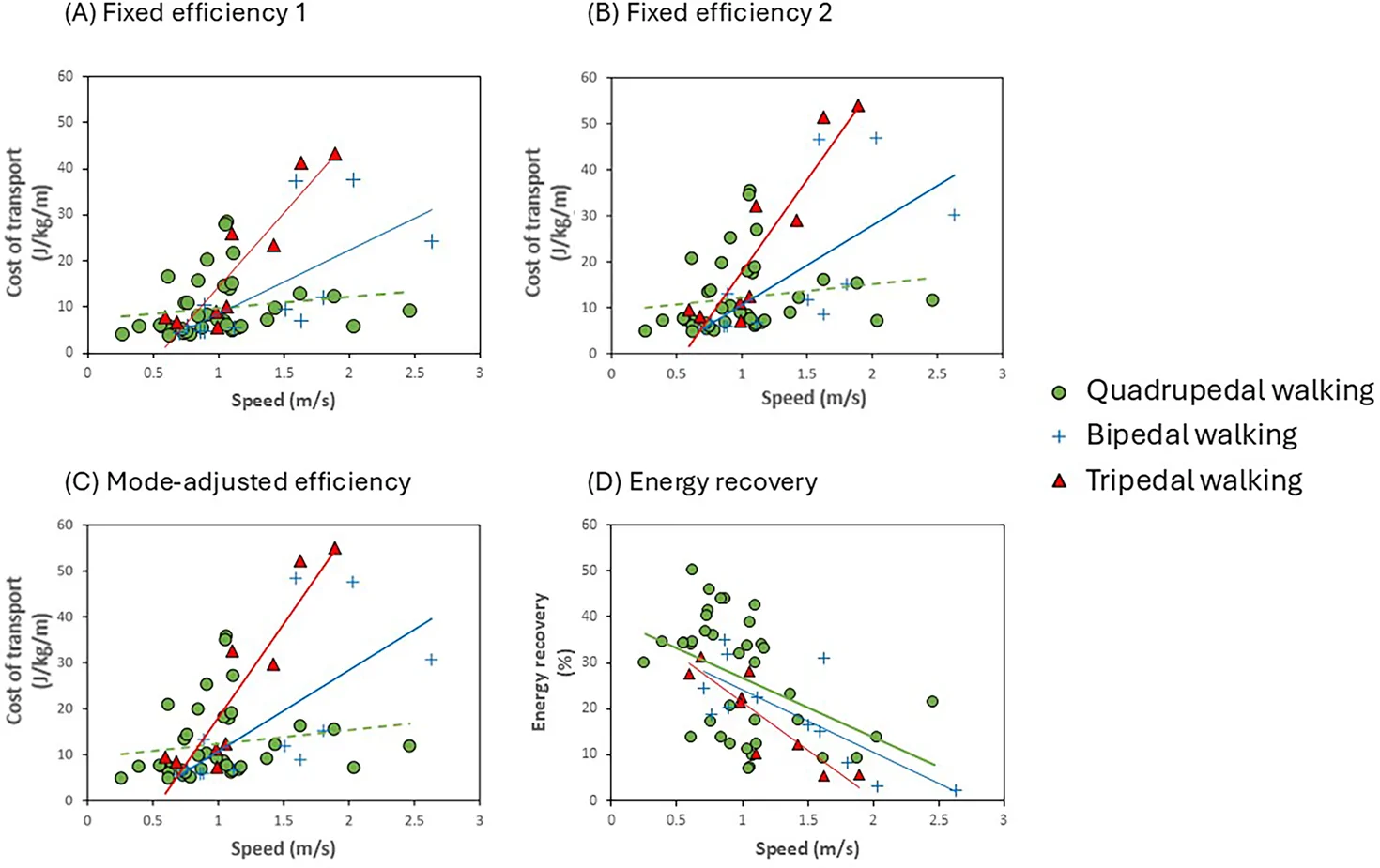
Net estimated metabolic cost of transport versus speed with fixed efficiency values 1 (A), 2 (B) and mode-adjusted values (C), and energy recovery versus speed (D) for quadrupedal (green circles), tripedal (red triangles) and bipedal walking (blue crosses).

Regarding vertical ascent, our values are lower than humans (26–32 versus 40.1 J kg⁻¹ m⁻¹, respectively; table 1; see [66]) and orangutans (approx. 39 J kg⁻¹ m⁻¹; estimated from table 1 in [38]), but match chimpanzees (approx. 24–34 J kg⁻¹ m⁻¹; estimated from [67]). Although mass-specific climbing metabolic costs are broadly constant across primates of varying body sizes (e.g. [18,68,69]), no 1–70 kg species has been directly measured. Furthermore, the substrate used during climbing and its properties probably influence energy costs, as they are probably to affect the difficulty of the movement (see [14] in humans; [41] in bonobos). For instance, climbing natural trees may be (mechanically) easier because animals can access multiple (horizontal) branches and choose between alternative routes, compared with climbing standardized structures (e.g. rubber tubes or ropes) typically used in respirometry studies (e.g. [14,18]). In addition, Kozma & Pontzer [66] suggested that climbing velocity is directly associated with gross energy expenditure. In our study, bonobos climbed roughly five times faster than humans (0.86 versus 0.16 m s⁻¹, respectively; table 1) [66], suggesting that speed may indeed play a key role in reducing gross climbing costs. Although we could not estimate postural costs and internal mechanical work in this study, bonobos’ morphological adaptations in their hands and shoulders (e.g. [42,70]) probably enable them to maintain more economical suspensory postures than humans, further lowering their energetic expenditure during climbing.

**Table 1.** Mean locomotor performance parameters per locomotor mode.

					metabolic cost of transport (J kg^−1^ m^−1^)		
modes	distance (m)	speed (m s^−1^)	mechanical CoT (J kg^−1^m^−1^)	recovery (%)	fixed efficiency 1	fixed efficiency 2	mode-specific efficiency
bipedal walk	2.56	1.37	2.91	19.10	13.61	16.92	17.27
vertical descent	1.56	0.94	1.45	58.48	10.54	12.97	10.79
vertical ascent	1.81	0.86	6.09	18.33	25.88	32.28	26.91
leap	2.13	1.26	4.22	22.70	18.91	23.43	19.52
quadrupedal walk	4.65	1.00	1.99	26.76	9.82	12.19	12.32
quadrupedal run	5.69	2.81	2.02	14.68	9.99	12.40	10.33
suspensory locomotion	1.71	1.16	1.66	51.33	10.36	12.78	8.46
tripedal walk	2.53	1.15	4.00	18.28	19.21	23.86	24.24

The percentage of recovery was higher during vertical descent and suspensory locomotion, but low in other modes, and decreased with speed during quadrupedal, bipedal and tripedal walking (*r* = –0.45, *r* = –0.76, *r* = –0.88, *p*< 0.01, respectively; figure 3D). Interestingly, net estimated metabolic energetic costs of suspensory locomotion matched quadrupedal walking. Moreover, branch recoil was not accounted for in this study, even though it may allow bonobos to reduce energy expenditure in arboreal contexts (see [38]). As previously observed [14,71], the most energetically expensive mode was vertical ascent, followed by leaping and tripedal walking. Speed variation was limited except during quadrupedal running (figure 2B). Overall, arboreal locomotion does not inherently impose a major energetic penalty compared with terrestrial quadrupedal walking, contradicting our hypothesis. Vertical ascent is the most costly component (see [14,38]), and minimizing height variation during daily activities could represent a relevant behavioural strategy for reducing overall energetic costs; however, energy minimization may not be the primary objective in natural habitats (e.g. [40,72–74]).

### (b) Predicting locomotor costs from locomotor repertoires

To estimate the energetic cost of locomotion in bonobos under natural conditions, we applied our mode-specific cost values to field-reported locomotor repertoires: Susman *et al.* [55], Doran [56] and Ramos [57], weighting by the average distance travelled per mode (in this study). For example, 5% of time spent walking quadrupedally within the locomotor repertoire does not represent the same distance travelled as 5% of time spent climbing. For each repertoire, we estimated costs for three hypothetical daily distances: 0.4, 2 and 6 km [75]. Note that Jang *et al.* [76] showed that chimpanzees travel 4.03 km on average per day, with a large range of variation, from 1.11 to 14.16 km. The previous estimations made on other sites show that chimpanzees travel from 2.1 to 4.2 km per day [77–79], which is similar to the range observed in bonobos [75].

For the locomotor repertoire described by Susman *et al.* [55], the estimated costs for travelling 0.4, 2 and 6 km are 61, 306 and 917 kcal, respectively, assuming the lowest muscle efficiency (i.e. the fixed efficiency value 2). Using the repertoire reported by Doran [56] results in similar estimates: 63, 314 and 941 kcal. By contrast, the locomotor repertoire described by Ramos [57], with more terrestrial quadrupedal walking, resulted in lower overall costs of 48, 240 and 720 kcal, respectively, probably owing to the more limited amount of climbing.

To contextualize these costs, Pontzer *et al.* [65] reported that bonobo daily energy expenditure (DEE) is approximately 1767 kcal d^−1^ and chimpanzee DEE is approximately 2386 kcal d^−1^, but these values are probably to vary; for example, from 500 to 2500 kcal d^−1^ in bonobos [80]. DEE comprises basal metabolic rate, the thermic effect of food, fixed costs of body maintenance and the energy required for physical activities such as locomotion. Among these components, locomotion can be highly variable [26,81,82], yet its contribution should remain modest in habitual daily routines (approx. 5%–15% [17,83,84]). For instance, using Doran's [56] repertoire, locomotor activities represent roughly 4%, 18% and 53% of bonobo DEE for 0.4, 2 and 6 km daily travel, respectively, and approximately 3%, 13% and 39% of chimpanzee DEE. With Ramos's repertoire [57], these values are approximately 3%, 14% and 41% of bonobo DEE, and approximately 2%, 10% and 30% of chimpanzee DEE. Although the costs estimated for a daily distance of 6 km appear unsustainable routinely, occasional longer distances may occur under particular circumstances (e.g. seasonal variation in vital area use has been reported [85]; see also [81,82,86]). In summary, daily travel of 1–4 km thus appears plausible as a regular routine.

### (c) What would be the energetic consequences of increasing reliance on bipedal walking?

These results can be used to model evolutionary transitions from energetic and behavioural perspectives. For instance, reversing quadrupedal/bipedal proportions in Doran's [56] repertoire yields locomotor costs of 4%, 21% and 62% of bonobo DEE for 0.4, 2 and 6 km daily travel, respectively. Our initial hypothesis was that increasing bipedal proportion in the bonobo repertoire would result in a substantially higher net estimated metabolic cost of locomotion, one that would be too high to sustain on a daily basis. However, our findings do not support this. Even for bonobos, 2 km travel in forested or fragmented forest-savannah mosaics, including roughly 35% bipedalism (weighted by the average distance covered during bipedal bouts), would result in approximately 1.2 km of bipedal walking. This remains energetically plausible within their locomotor repertoire, suggesting that early hominins could have integrated bipedal walking alongside other locomotor modes, including arboreal locomotion. The key evolutionary step may have been a routine increase in travel distance beyond 2 km, driving selective pressure for bipedal efficiency. Although directly linking locomotor costs to DEE is challenging owing to compensatory mechanisms (e.g. [81,86]), establishing the range of locomotor costs among non-human primates provides a framework for modelling repertoire variation and understanding how shifts in behaviour may have influenced energy budgets in early hominins.

## Supplementary Material



## Data Availability

All raw data, MATLAB code and final tables are publicly available on the Open Science Framework [87]. Supplementary material is available online [88].
