# Supplementary material for: Energy expenditure of bonobos across their locomotor repertoire
Source: Biol Lett. 2026 Oct 14;22(10):20260427. doi: 10.1098/rsbl.2026.0427 (PMC13682554; doi:10.1098/rsbl.2026.0427)
Supplement: Supplementary file 1 [file rsbl.2026.0427.zip › rsbl.2026.0427/rsbl20260427_si_001.docx]

**Supplementary material – *Biology Letters***

**“Energy expenditure of bonobos across their locomotor repertoire”**

By François Druelle, Jean-Pascal Guéry, & Victor Narat

A. ***Distribution of selected locomotor bouts across age-sex classes***

In our analysis, juveniles exhibited a variety of locomotor modes, whereas adult males and females were primarily observed engaging in ground-based behaviors such as walking and running. The number of bouts analyzed per locomotor mode ranged from a minimum of 9 (tripedal walking and leaping) to a maximum of 38 (quadrupedal walking).

B. ***Sensitivity analysis of the BCoM position in bonobo across different postures***

C. **Effect of the 4Hz low-pass filter on raw data: Visual assessment and supplementary notes**

The context of our study differs from the conventional approach for calculating external mechanical work across stride cycles, as our sequences are substantially longer and often include multiple locomotor modes. Consequently, we selected a cut-off frequency of 4Hz, which we deemed appropriate for sequences of all lengths (see figures above).

Nevertheless, it is important to note that the filtering process can significantly influence the results. Smoothing techniques inherently alter the signal to some extent (see Chapter 11 in Robertson *et al*., 2013). For instance, using a 7Hz filter instead of the 4Hz filter applied in this study would increase the cost of transport (assuming a fixed efficiency value of 1) by 44% for bipedal walking, 40% for climbing down, and 13% for climbing up. On average, this could result in a 50% increase in overall costs across all locomotor modes. That said, such a higher cut-off frequency would likely be inappropriate for the signal-to-noise characteristics observed in our data.

Robertson, D. G. E., Caldwell, G. E., Hamill, J., Kamen, G., & Whittlesey, S. (2013). *Research methods in biomechanics*. Human kinetics.
